# Treat Me Well or Will Resist: Uptake of Mobile Genetic Elements Determine the Resistome of *Corynebacterium striatum*

**DOI:** 10.3390/ijms22147499

**Published:** 2021-07-13

**Authors:** Benjamin Leyton, Juliana Nunes Ramos, Paulo Victor Pereira Baio, João Flávio Carneiro Veras, Cassius Souza, Andreas Burkovski, Ana Luíza Mattos-Guaraldi, Verônica Viana Vieira, Michel Abanto Marin

**Affiliations:** 1 Scientific and Technological Bioresource Nucleus (BIOREN), Universidad de La Frontera, Temuco 4811230, Chile; leyton.bl@gmail.com; 2 Carrera de Bioquímica, Departamento de Ciencias Químicas y Recursos Naturales, Universidad de La Frontera, Temuco 4811230, Chile; 3 Laboratório Interdisciplinar de Pesquisas Médicas, Instituto Oswaldo Cruz, Fundação Oswaldo Cruz—Fiocruz, Rio de Janeiro 21040-361, Brazil; jnr.uerj@gmail.com (J.N.R.); pvpbaio@gmail.com (P.V.P.B.); joao.veras@ioc.fiocruz.br (J.F.C.V.); veronicavianavieira@hotmail.com (V.V.V.); 4 Laboratory of Diphtheria and Corynebacteria of Clinical Relevance, Faculdade de Ciências Médicas, Universidade do Estado do Rio de Janeiro—LDCIC/FCM/UERJ, Rio de Janeiro 20550-170, Brazil; cassiusideal@gmail.com (C.S.); aguaraldi@gmail.com (A.L.M.-G.); 5 Department of Biology, Professur für Mikrobiologie, Friedrich-Alexander-Universität Erlangen-Nürnberg, Staudtstrasse 5, 91058 Erlangen, Germany; andreas.buskovski@fau.de

**Keywords:** *Corynebacterium striatum*, multidrug resistance mechanisms, insertion sequences, transposons, Tn5432, MLS phenotype, resistome, emerging pathogen, pTP10 plasmid, antibiotic resistance genes

## Abstract

*Corynebacterium striatum*, a bacterium that is part of the normal skin microbiota, is also an opportunistic pathogen. In recent years, reports of infections and in-hospital and nosocomial outbreaks caused by antimicrobial multidrug-resistant *C. striatum* strains have been increasing worldwide. However, there are no studies about the genomic determinants related to antimicrobial resistance in *C. striatum*. This review updates global information related to antimicrobial resistance found in *C. striatum* and highlights the essential genomic aspects in its persistence and dissemination. The resistome of *C. striatum* comprises chromosomal and acquired elements. Resistance to fluoroquinolones and daptomycin are due to mutations in chromosomal genes. Conversely, resistance to macrolides, tetracyclines, phenicols, beta-lactams, and aminoglycosides are associated with mobile genomic elements such as plasmids and transposons. The presence and diversity of insertion sequences suggest an essential role in the expression of antimicrobial resistance genes (ARGs) in genomic rearrangements and their potential to transfer these elements to other pathogens. The present study underlines that the resistome of *C. striatum* is dynamic; it is in evident expansion and could be acting as a reservoir for ARGs.

## 1. Introduction

A growing number of case reports have demonstrated the relevance of *Corynebacterium* spp. as etiologic agents of various infectious processes in both immunocompromised and immunocompetent patients. In addition to the etiologic agents of classic and zoonotic diphtheria, at least 50 diphtheria toxin (DT)-producing and non-DT-producing *Corynebacterium* species have been recognized as medical, veterinary, or biotechnologically relevant, including members of human microbiota [1,2,3]. Clinical implications in infected patients due to the expression of multidrug-resistance (MDR) profiles and virulence mechanisms have been increasingly observed for varied non-DT-producing *Corynebacterium* species, including *Corynebacterium jeikeium*, *Corynebacterium urealyticum*, *Corynebacterium amycolatum*, *Corynebacterium pseudodiphtheriticum*, *Corynebacterium afermentans*, and *Corynebacterium striatum*. Geographical variations in the frequency of non-DT-producing *Corynebacterium* spp. as an etiologic agent of a range of type infections have been reported [1,4,5,6]

In the past, *C. striatum* strains were described only as saprophytic microorganisms colonizing the skin and nasal mucosa of healthy individuals and unable of causing disease [7]. However, different studies have increasingly recognized *C. striatum* as the etiologic agent of various infections with signs and symptoms ranging from mild to severe to fatal outcomes, including bacteremia, endocarditis, meningitis, osteomyelitis, arthritis, corneal infection, and sinusitis [8,9]. Moreover, cases of infection of the respiratory tract, surgical wounds, skin lesions, or the eye, genital and urinary tract due to *C. striatum* strains have been also reported [10,11,12]. Previous studies verified nosocomial spread of MDR *C. striatum* strains, especially in patients of intensive care units and surgical wards making use of medical devices or with prolonged exposure to broad-spectrum antimicrobials [13,14,15]. The use of indwelling medical devices along with other clinical practices has been demonstrated to facilitate colonization, infection of skin lesions and/or mucosal surfaces with subsequent invasive dissemination by *C. striatum* strains [16,17,18,19,20,21]. Infections caused by *C. striatum* are also associated with sternal surgical wound wires [22]. Nosocomial infections and outbreaks caused by *C. striatum* are mainly found the respiratory tract, notably due to mechanical ventilation support as a risk factor in different countries [22,23,24,25,26]. During a nosocomial outbreak in Rio de Janeiro (RJ), Brazil, cases of ventilator-associated pneumonia and tracheobronchitis in addition to catheter-related sepsis caused by MDR *C. striatum* strains were verified [1]. Recently, a review study and analysis of clinical–epidemiological and microbiological features of a total of 218 reported research studies during a 44 year period (1976 to 2020) verified that MDR and multidrug susceptible (MDS) *C. striatum* strains were identified in 254 reported cases as nosocomial and community pathogens worldwide [25].

*C. striatum* strains expressing varied MDR profiles have been identified as the etiologic agent of various types of both nosocomial and non-healthcare associated infections reported in industrialized and developing countries [25,27]. Recently, resistance to antiseptic and disinfectants such as glutaraldehyde (GA) has also been reported for *C. striatum* [28]. Accordingly, medical surveillance programs should include control strategies to decrease potential risk factors of nosocomial infections and outbreaks due to *C. striatum*. Therefore, investigation of clinical, epidemiological, and virulence features is necessary, especially for preventing and disseminating *C. striatum* strains expressing MDR profiles among nosocomial patients worldwide. The emergence of MDR strains and heterogeneity of MDR profiles, in addition to the fact that different clones may be responsible for nosocomial outbreaks, have been problems of major concern [1,12,13,22,27,29].

In Brazil, the analysis by pulsed-field gel electrophoresis (PFGE) of *C. striatum* strains indicated the presence of four different PFGE types isolated from varied clinical specimens of patients in different hospital wards during a nosocomial outbreak: MDR *C. striatum* strains of PFGE-types I to II and non-MDR *C. striatum* strains of PFGE-types III to IV. MDR *C. striatum* strains expressing PFGE-type I and type II profiles were found predominant and mainly isolated from tracheal aspirates of patients undergoing endotracheal intubation procedures, while only two MDR *C. striatum* strains expressing PFGE-types I and II were isolated from blood samples [1]. However, a subsequent increase in the number of the bloodstream and catheter-related infections caused by *C. striatum* strains was verified in the Brazilian hospital. Further investigation revealed the permanence of MDR *C. striatum* strains PFGE-types I and II in nosocomial wards and predominance of PFGE- type I among patients with hematogenic infections and the presence of particular and genetically related PFGE-profiles. Therefore, virulence potential and invasive properties of *C. striatum* strains belonging to 10 particular or genetically related profiles expressing heterogenic MDR were documented by their ability to cause bloodstream and catheter-related infections within the nosocomial environment. Moreover, the high level of MDR associated with biofilm formation capacity observed for invasive *C. striatum* strains was also found as an issue of concern. Therefore, data suggested a relationship between biofilm formation ability, changes in genes, and increased resistance to drugs for *C. striatum* [12,27,28].

The pathogenicity potential of *C. striatum* strains due to a virulence mechanism of a multifactorial nature and the influence of environmental conditions has been investigated in different regions. Distinct studies verified the ability of biofilm formation as a significant factor affecting the clinical relevance of *C. striatum*. The ability of biofilm formation and survival on abiotic surfaces was confirmed for MDR and MDS *C. striatum* clinical isolates obtained from patients presenting varied types of infections [12,27,30,31].

Whole-genome sequencing (WGS) has been an essential tool for understanding molecular epidemiology, global transmission, and virulence mechanisms of pathogens. Although WGS has been used for *C. striatum* investigation of genomic features of MDR and non-MDR clinical isolates, further studies remain necessary to study strain-dependent genomic elements related to the acquisition of resistance to antimicrobial agents [16,32,33,34].

At the time of finishing this review, there were no studies on the antimicrobial resistance genes repertoire in *C. striatum*, possibly because the literature is scarce and related to specific countries. This review aims to compile bibliographic information and information derived from publicly available genomic data to understand the epidemiology of multidrug-resistant *C. striatum* and identify genomic determinants of this resistance. Thus, in this review, we describe the intrinsic and extrinsic factors related to the acquisition of antimicrobial resistance. Moreover, we detail the genomic context of these elements, the relationship between them and their impact on the conformation of the resistome in *C. striatum*. Finally, we discuss the role of mobile genetic elements in the acquisition, maintenance and dispersal of ARGs, as well as future challenges and actions related to the resistome of *C. striatum*.

## 2. Genome Organization of *C. striatum*

According to the genomes available in the NCBI Pathogens Database (https://www.ncbi.nlm.nih.gov/pathogens/), there are 131 genomes (accessed on 31 October 2020) of *C. striatum* available. Genomes include complete, draft, and raw data genomes from the USA, China, Brazil, Japan, and, more recently, genomes from Germany and Denmark. The genome length is ~2.8 Mb, and the GC content is around 59% [34]. Genomic data of *C. striatum* from NCBI shows three plasmids in *C. striatum*, pCs-Na-1, pCs-Na-2, and pTP10. The pTP10 plasmid has been reported in *C. striatum* M82B [35] and its presence in other genomes been studied [34]. The presence of viral genomes in the *C. striatum* has not been adequately studied, but recently a study identified a phage species from the *Siphoviridae* family in *C. striatum* [36]. Pangenomics studies have not yet been reported. More deposited genomes and genomic analyses are needed to better understand the genome structure and evolutionary dynamics of *C. striatum*.

## 3. *C. striatum* Resistome

The resistome is the total repertoire of genes that contribute to resistance in bacteria, including ARGs. Different antibiotic resistance mechanisms are described in isolates of clinical importance, which have been grouped into intrinsic and extrinsic mechanisms, referring to persistence and resistance mechanisms, respectively [37]. The intrinsic mechanisms are natural phenomena present in all the bacteria; they are generally obtained by regulating membrane permeability and non-specific efflux pumps. In addition, chromosomally encoded elements have been attributed to this phenotype [38]. Conversely, the extrinsic or generally acquired mechanisms are obtained via the horizontal transfer of mobile elements. These mechanisms include specific efflux pumps, antibiotic modifying enzymes, or modifying the antibiotic target using genes acquired by horizontal transfer [37]. In the genus *Corynebacterium*, several mechanisms of resistance to both intrinsic and extrinsic antimicrobials have been described. Resistance to fluoroquinolones and daptomycin in *Corynebacterium* spp. are examples of an intrinsic mechanism associated with point mutations in the *gyrA* and *pgsA2* genes, respectively [39,40].

**Figure 1:**
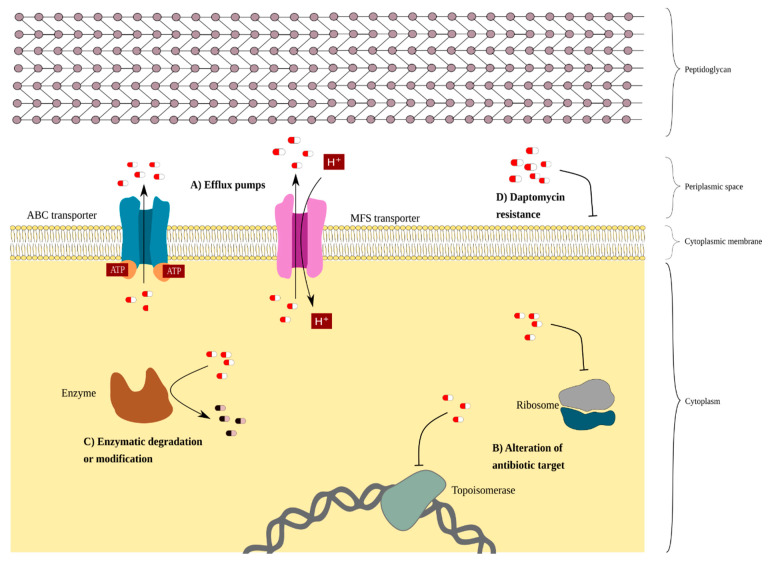
A schematic representation of the drug-resistance mechanism of *C. striatum*. Antibiotics are represented as pill shapes in white and red. (**A**) The efflux pumps transport phenicols, tetracyclines, and beta-lactams out of the membrane cell. The blue complex protein represents an ABC transporter encoded by the *tetA/B* genes responsible for transporting tetracyclines and beta-lactams. The pink complex protein represents a specific MFS transporter for chloramphenicol. (**B**) Altering the target of the antibiotic is a form of resistance. Alteration of drug target due to mutations in *gyrA* prevent the binding of fluoroquinolones to topoisomerases. Resistance to macrolides is due to the modification of the target through the methylation of the 23S subunit of the ribosome, caused by the *erm* gene. Similarly, the *tet(W)* gene encoding a ribosomal protective protein, TET(W), confers resistance to tetracyclines. (**C**) Resistance to aminoglycosides is given by the enzymatic modification (phosphorylation or adenylation) of these antibiotics, preventing the binding to the ribosome. Beta-lactam resistance is due to B-lactamases encoded by the *bla* and *ampC* genes, which degrade beta-lactam antibiotics. (**D**) Daptomycin resistance is due to the mutation in the *pgsA2* gene causing a phosphoglycerol (PG) deficiency in the membrane; this PG deficiency prevents the binding of daptomycin to the membrane.

On the other hand, the presence of plasmids in *Corynebacterium* spp., although rare [41], is associated with the presence of ARGs. Examples of these plasmids in corynebacteria are: pJA144188 in *C. resistens* [42]; pNG2 in *C. diphtheria* [43]; and pTP10 in *C. striatum*. In *C. striatum*, resistance to fluoroquinolones and daptomycin has been described, which are intrinsic mechanisms. Resistance to other antimicrobials that have likely been acquired by horizontal gene transfer has also been described (Figure 1). Next, different resistance mechanisms in *C. striatum* are outlined.

### 3.1. Intrinsic Resistance in C. striatum

Fluoroquinolones are a group of synthetic broad-spectrum antimicrobials in Gram-negative and Gram-positive bacteria. Fluoroquinolones inhibit bacterial type II topoisomerases such as DNA gyrase and topoisomerase IV, which are crucial to unwinding and transcribing bacterial DNA [44]. Fluoroquinolone resistance mechanisms include the alteration of membrane permeability, efflux, inactivation by acetylation and, the most common mechanism, mutations at the site of action of the antibiotic in topoisomerases [37,38]. In *C. striatum*, several studies have described mutations in the quinolone resistance-determining regions (QRDR) in the *gyrA* gene [12,16,17,34,45,46,47]. Sierra and co-workers (2005) [46] reported mutations for positions 87 and 91 in the peptide sequence of GyrA. Later investigations performed by Ramos and co-workers (2018 and 2020) [34,45] showed a preference of *C. striatum* isolates to mutate from Ser87Val and less often from Asp91Asn or Asp91Ala. Alibi et al. (2017) [47] reported mutations of Ser87Phe and Asp91Ala or Asp91Gly, while Drogomirescu et al. (2020) [20] showed that *C. striatum* strains increased their resistance to fluoroquinolones when the mutation occurred from Ser87Phe and Asp91Ala. The change from Ser87 and Asp91 to non-polar amino acids suggests the –OH group from Ser87 and the –COOH group from Asp91 play an important role in binding the quinolone antibiotic. Interestingly, Nudel et al. (2018) [16] found new mutated positions in their *C. striatum* tested strains, i.e., Ser95Thr, Asp94Ala, Glu88Ala and Asp87Gly; however, they follow the same logic to switch polar for non-polar amino acids (except position 95), thus reducing their fluoroquinolone affinity.

Daptomycin is a calcium-dependent lipopeptide antibiotic produced by *Streptomyces roseosporus*. It is generally used to treat serious infections caused by Gram-positive bacteria. Daptomycin could act as a temporary ionophore in the membrane by forming a complex with phosphatidylglycerol (PG) and calcium ions, bringing about depolarization and a subsequent ion leakage. An ion leakage can have several consequences, including an osmotic imbalance and metabolite uptake deficiency. However, the bactericidal mechanisms of daptomycin are not yet entirely clear [48]. Initially, daptomycin resistance had been only reported in the genus of corynebacteria in *C. jeikeium* [6]; later isolates of daptomycin-resistant *C. striatum* were found [19,40,49,50,51,52]. Moreover, a recent study showed that several corynebacteria species could rapidly develop daptomycin non-susceptibility, including HLDR (high-level daptomycin resistance), after a short daptomycin exposure period [53].

The daptomycin-resistance mechanisms in *C. striatum* are due to the loss of mutation function in the *pgsA2* gene. This gene encodes for phosphatidylglycerol synthase A, responsible for synthesizing PG from diphosphate diglyceride (CDP-DAG) in the PG synthesis pathway [40,51,54], which leads to a significant loss of PG in the membrane of *C. striatum*. A study showed that the loss of PG in the membrane could be lethal in *Bacillus subtilis* and therefore PG is vitally essential to membrane integrity [55]. Nevertheless, Goldner et al. (2018) [40] demonstrated that the HLDR phenotype in *C. striatum* was sufficient with the loss of *pgsA2* gene function. Namely, the HDLR phenotype in *C. striatum* does not involve additional mutations or changes in the transcription levels of genes biosynthetically linked to *pgsA2*, which suggests that *C. striatum* is an even more persistent and adaptable bacterium than *B. subtilis*. The mutations found in *pgsA2* can include conserved sites, active sites, substrate binding sites, and stop codon mutations [40,51]. Moreover, the HLDR phenotype has been easily produced in vitro and during treatment in patients [40].

Although no further gene mutations have been reported to date (in addition to *gyrA* and *pgsA2*) that confer a resistant phenotype, the rapid adaptation to daptomycin tolerance and growing resistance to fluoroquinolones demands that more studies and focused monitoring be performed on the emergence of the persistence of intrinsic mechanisms associated with antibiotic resistance in *C. striatum*.

### 3.2. Extrinsic Resistance Associated with Mobile Elements

In corynebacteria, different ARGs associated with mobile genetic elements, including plasmids, integrons (In), insertion sequences (IS), and transposons (Tn), have been reported [39,43,56,57]. In *C. striatum,* resistance is mainly determined by Tn, IS, and plasmids. Interestingly, a 38–66 kb region was found between the chromosomal genes *dppD* and *cgrA/B* in *C. striatum*. This region contains all the mobile elements with their associated ARGs, and this region is probably an access point to the chromosome for horizontal transfer vectors such as plasmids [16]. Of the three known plasmids in *C. striatum* (pCs-Na-1, pCs-Na-2 and pTP10), only pTP10 is known to confer resistance. The pTP10 plasmid is a multidrug resistance mosaic reported in 1983 by Kono et al. [58] on the *C. striatum* M82B strain initially poorly characterized as *C. xerosis*. The size of the pTP10 plasmid is 51,904 bp and contains 47 ORFs organized in eight regions, of which two are regulatory regions, and six are transporter regions of ARGs [35].

#### 3.2.1. Resistance Due to Tn5432

The MLS phenotype (resistance to Macrolides, Lincosamides, Streptogramin) is a mechanism frequently observed in coagulase-negative staphylococci [59,60]. It is also widely seen in corynebacteria of clinical importance, such as *Corynebacterium diphtheriae*, *Corynebacterium xerosis*, *C. jeikeium*, *C. pseudodiphtheriticum*, *C. amycolatum*, *C. urealyticum* [61,62,63,64,65], and *C. striatum*. The MLS phenotype is associated with the *erm(X)* gene transported by transposon Tn5432 [35].

**Figure 2:**
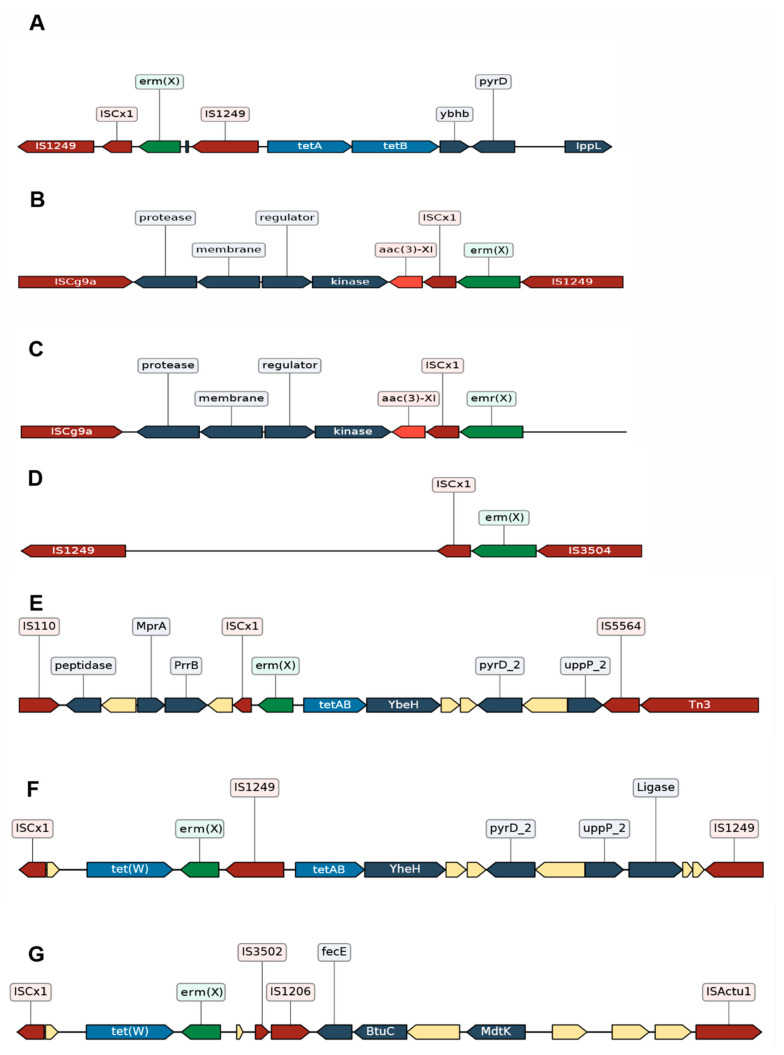
Genomic context of Tn5432. Illustrated sequences from the genomic context of Tn5432 found in studies by Nudel et al., 2018; Tauch et al., 2000; Wang et al., 2019. The mobile elements are represented in red (IS and Tn), hypothetical proteins are depicted in the empty boxes, the genes directly unrelated to the resistance to antibiotics are in dark blue. (**A**) Tn5432 flanked by two IS1249 elements transports the *erm(X)* gene (in green) to its corresponding right, region II of the pTP10 plasmid of *C. striatum* that contains the *tetA/tetB* genes (in light blue). (**B**–**D**) Regions with the same genomic context as Tn5432 found in strains in Boston, USA [16], but in addition to the *erm(X*) gene, this region contains the *aac(3)-XI* gene (in orange). (**E**–**G**) Regions with the same genomic context as Tn5432 found in Beijing, China [33]. The *erm(X)* gene, the *tetA/tetB* genes, and the *tet(W)* gene can be seen. (**E**) Note that it is the same genomic context; if *erm(X)* is located in the center, on the right can be seen segment II of the pTP10 plasmid in the isolates from China, and on the left the same segment of the isolates from the USA although annotated as protease, membrane, regulator, and kinase. (**B**–**D**) correspond to the genes annotated as *peptide*, *hypothetical protein* (associated with membrane), *MprA* (a regulator) and *PrrB* (a kinase). The representation of the sequences was done with the DNA Features Viewer tool [68] by using as references the accession numbers AF024666.2 (**A**), GCA_002804085.1 (**B**), GCA_002803965.1 (**C**), SRR5120233 (**D**), GCA_015945985.1 (**E**), GCA_015946165.1 (**F**), GCA_015946545.1 (**G**).

The transposon Tn5432 is the first region of the pTP10 plasmid, and it is a composite transposon. A composite transposon consists of two inverted repeats from two separate identical or related insertion sequences (IS) moving together as one unit and carrying the DNA between them (e.g., an ARG) [66]. Tn5432 is flanked by two identical insertion sequences (IS1249) and carries the *erm(X)* gene [35] (Figure 2A) responsible for resistance to lincosamides and macrolides such as clindamycin and erythromycin. There are different locations of the *erm(X)* gene and Tn5432; in both *C. xerosis* and *C. diphtheriae,* they are found in their plasmids, while in *C. jeikeium* and *C. striatum,* they are found in their chromosomes (53). Tn5432 carries ISCx1, which is interesting since ISCx1 can be evidence of a “genomic scar” of Tn5432 because it is a truncated IS element (due to a stop codon). We observed the presence of the ISCx1 element near the *erm(X)* gene in different studies conducted in the USA, Brazil, and China (Figure 2) [16,33,34]. Nevertheless, these studies report rearrangements for Tn5432. Nudel et al. (2018) [16] reported a genomic context similar to Tn5432 flanked by IS1249, ISCg9a-like, IS3504-like (Figure 2B–D), and IS407. Furthermore, in the Tn5432 context, they found the presence of the *aac(3)-XI* gene, responsible for resistance to aminoglycosides such as kanamycin, gentamicin, and streptomycin, among others. On the other hand, Wang et al. (2019) [33] reported a genomic context similar to Tn5432 (Figure 2E–G), but with rearrangements different from those presented by Nudel et al. (2018) [16]. Nevertheless, they have shown the ISCx1 and *erm(X)* together with the *tet(W)* gene and also the *tetAB* regulator, *pyrD* and *ippL-like* genes were present next to *erm(X)* and ISCx1 (Figure 2E,F). These genes correspond to the second region of the pTP10 plasmid reported by Tauch et al. (2000) [33], and a co-integration of this segment has been described together with Tn5432 in *Corynebacterium glutamicum* [35]. The presence of region II, the *erm(X)* gene, and ISCx1 in these studies suggests that in addition to resistance to lincosamides and macrolides, Tn5432 confers tetracycline resistance, which a past recombination event could have triggered. Eady et al. (2000) [62] evaluated the co-resistance of erythromycin and tetracyclines in different coryneform and Gram-positive cocci on the skin’s surface, and they found that most of the isolated bacteria, including *C. striatum*, presented resistance to tetracyclines as well as resistance to erythromycin. Interestingly, the region in Figure 2E comprising genes assigned as *peptidase*, *hypothetical protein*, *mprA*, and *prrB* is syntenic to the region from Figure 2B,C (genes assigned as *protease*, *membrane*, *regulator* and *kinase*). This observation further reinforces that they are in the same genomic context.

According to the ISfinder database [67], IS1249, the insertion sequence flanking Tn5432, has been found in other genomes of the genus, such as *C. urealyticum* and *C. jeikeium*, and other bacteria such as *Propionibacterium acnes* and *Bifidobacterium thermophilum*. This scenario indicates that these elements are potential propagators of resistance at the interspecies level.

#### 3.2.2. Acquired Resistance Genes for Chloramphenicol (*cmx*) and Aminoglycosides (*strA* and *strB*) Are in the Same Genomic Context

**Figure 3:**
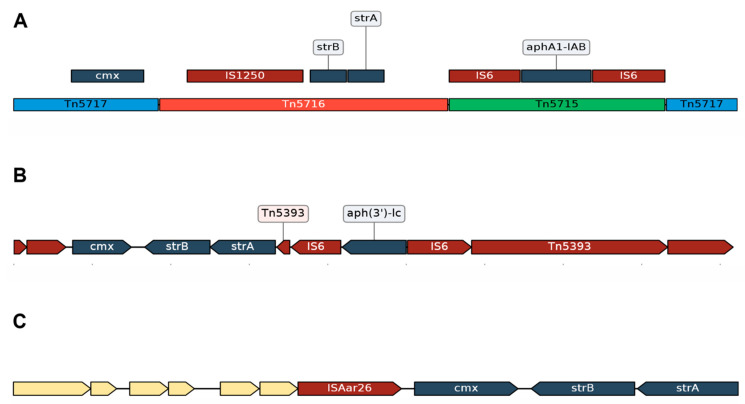
Genomic context of *cmx*, *strA* and *strB* genes. Antibiotic resistance genes are represented in dark blue, the mobile elements in dark red and the hypothetical proteins in the empty boxes. (**A**) Segment belonging to the pTP10 plasmid of *C. striatum* was documented by Tauch et al., 2000. The segment contains the transposons Tn5717 (transporting the *cmx* gene), Tn5716 (transporting the *strB-strA* genes) and Tn5715 (transporting the *aphA1-IAB* gene). (**B**) Segment belonging to the isolates from Boston, USA [16]. The region shows the *cmx*, *strB-strA* genes near a composite transposon (transporting the *aph(3′)-lc* gene) flanked by two IS26 similar to Tn5715; in addition, the absence of the IS1250 element can be observed. Similarly, the isolates from Beijing, China [33] (**C**) showed the *cmx* and *strB-strA* genes are close and without the IS1250 element, in the same sense as the isolates from the USA. The representation of the sequences was done with the DNA Features Viewer tool [68], considering the accession numbers AF024666.2 (**A**), GCA_002803965.1 (**B**), GCA_015946165.1 (**C**).

In *C. striatum*, the only mechanism of resistance to chloramphenicol is associated with the *cmx* gene, which codes for an efflux pump. On the other hand, *strA* and *strB* confer the resistance to the aminoglycoside streptomycin. Initially, the *cmx*, *strA-strB*, and *aphA1-AB* genes were carried by Tn5717, Tn5716, and Tn5715 (Figure 3A), respectively, in the pTP10 plasmid [35]. These transposons correspond to IV, V, and VI from this plasmid region. The organization of these regions is interesting, where Tn5717 is interrupted by Tn5716, which is interrupted by Tn5715. A similar ordering was found in the studies by Nudel et al., 2018 (Figure 3B) [16], in which the *cmx*, *strB*, and *strA* genes plus an element similar to Tn5715 composed of IS26 flanking the *aph(3′)-lc* gene can be seen together. Wang et al., 2019 [33], showed that the *cmx*, *strB*, and *strA* genes were found in the same genomic context transported by ISAar26 (Figure 3C) and were widely distributed in a cluster in their analyses. All this suggests that it is the same genomic context, and they were acquired together; for the rest, it has not been proven that these genes have been acquired independently. However, it is known that pTP10 transports Tn5564, which contains only the *cmx* gene and IS1513. Ramos et al. (2018) [34] assessed the presence of the pTP10 plasmid and demonstrated that a large part of the plasmid was found in the genome (*C. striatum* 2308), including Tn5564. Knowing that chloramphenicol resistance could be due to other intrinsic and extrinsic mechanisms, future studies to identify these will be needed for *C. striatum*.

#### 3.2.3. Different Genomic Contexts of the *tet(W)* Genes Suggests Possible Recombination Events

The *tet(W)* gene product eliminates the inhibitory effect of tetracycline on protein synthesis through the non-covalent modification of the ribosomes. The gene is not transported by pTP10, suggesting another recombination event beyond pTP10 (or this plasmid acquired this gene in later events). It has been reported that IS3504 carried the *tet(W)* gene in isolates from the USA [16], but it could also be transported by IS3503 and IS3502 [33]. According to the ISfinder database [67], both IS3503 and IS3502 are part of *C. jeikeium*, and it has been transported to its plasmids and to *C. urealyticum*. On the other hand, IS3504 was described in the pNG2 plasmid of *C. diphtheria* [35] and the pJA144188 plasmid of *C. resistens* [42]. The *tet(W)* gene was first described for corynebacteria in plasmid pJA144188 of *C. resistens*. The gene was described in a genomic context similar to how it presents in *C. striatum*, with a close IS3504 element, as the studies by Nudel et al. (2018) [16] described it, and close to the *erm(X)* gene as was found in the studies by Wang et al., 2019 [33].

The genomic contexts associated with transposons Tn5432, Tn5717, Tn5716, and Tn5715 suggest that pTP10 underwent past recombination events with the chromosome, constituting a portion in the chromosome open to new recombination events. New realignments of the transposons can explain this, the appearance of the *tet(W)* and *aac(3)-XI* genes, and the diversity of insertion sequences. Therefore, we propose that insertion sequences play a fundamental role in the continuous formation and evolution of the resistome of *C. striatum*.

#### 3.2.4. Impact of the Insertion Sequences in the Resistome of *C. striatum*

Insertion sequences (IS) are small mobile elements; they typically contain a gene that codes for a transposase (*tnp*). IS elements are grouped according to the motives of the active site and according to their recombination mechanism. Traditionally, IS elements do not transport passenger genes, but they can move ARGs as part of a composite transposon, which is a region flanked by two copies of the same IS element (as is the case of the Tn5432; See Figure 2A) or a related one, which can be mobilized as a unit [66]. IS elements and Tn can be mobilized by themselves in the same DNA molecule or between different DNA molecules by recombination (conjugation, transduction, and transformation). *C. striatum* contains several IS elements (Table 1) related to the ARGs, grouped mainly in the IS3 and IS256 families. However, it also has elements from the IS6 and IS110 families. Regarding the origin of these elements, since most seem to be in Actinobacteria, it suggests that these elements are preserved in the phylum and the other species of the genus *Corynebacterium* (Table 1). Thus, we postulate that the diversity of IS elements present in *C. striatum* could be reflected in the nosocomial environment where it coexists with the rest of the microbiota. The IS3 family is one of the largest, being widely distributed in nature. The IS256 family is abundant in the vaginal metagenome, and the IS6 family is abundant in the skin metagenome [69,70]. The IS6 family only shows associations with aminoglycoside resistance genes in *C. striatum* (Table 1), although there are no patterns of families of IS elements with some types of antimicrobials [70]. Alternatively, unlike other IS elements that have a “cut-out-paste” or “copy-out-paste” mechanism, IS6/26 has a replication transposition mechanism or “copy-in”, which involves events of reciprocal recombination by resolvase (co-integrates), which leads to rearrangements of the genome [69,71,72].

There are elements in the IS3 and IS256 families (apparently mostly in *C. striatum*) that can form hybrid promoters, which generate a promoter derived from two different sequences [73]. For the rest, it is known that a hybrid promoter can be more efficient than its parent promoters [74]; but, the hybrid promoters in some IS elements have been reviewed and seem to have a more random effect [73], being more or less efficient. Therefore, the resistance levels of *C. striatum* can be explained partly by the efficiency of the promoter of its IS elements. However, further research will be required on these elements in *C. striatum*.

In addition to the potential role of IS elements in the expression of ARGs, they may have a role in the grouping or concentration of genes through a selection process that could provide a common promoter [75]. The formation of operons and integrons would be advantageous to conserving the beneficial genes, in this case, ARGs, and would increase their intra- and intercellular mobility [70,75]. In other words, we postulate that the IS elements could contribute to the acquisition and maintenance of key genes for the adaptation and fitness of *C. striatum*, making it a potential reservoir for ARGs. This scenario could be explained due to the persistent exposure to antimicrobials, which select strains harboring ARGs and/or an improved association of IS with ARGs in *C. striatum*.

### 3.3. C. striatum Has Resistance Genes Probably Associated with Mobile Elements

*C. striatum* is resistant to several antimicrobials (Table 2), with penicillin being well known. In *C. striatum,* resistance to these beta-lactams has been associated with the *ampC* and *bla* genes which encode beta-lactam ring-modifying enzymes [47]. It has also been associated with non-specific efflux pumps encoded for the *tetA/B* genes [35]. In other corynebacteria such as *C. jeikeium*, *C. urealyticum,* and *C. resistens*, it has been noted that they harbor the *bla* gene, but they have not been associated with resistance to beta-lactams [47]. The *blaB* gene, which encodes for a beta-lactamase regulatory protein, has been noted in the studies by Wang et al. (2019) [33]; this suggests that *C. striatum* has the regulatory mechanism *bla* gene, needed for conferring beta-lactam resistance.

The resistance to sulfonamides may be due to the *sul* genes found in the studies by Wang et al. (2019) [33]. The *sul* gene confers sulfamethoxazole resistance (Table 2). According to the NCBI pathogens database, resistance genes such as *merA*, which confers mercury resistance, are annotated. In addition, there is the *qacEdelta1* gene, which confers resistance to quaternary ammonium compound biocides. Biocide resistance was reported and associated with biofilms by Souza et al. (2020) [28]. On the other hand, the *sul* gene and the *qacEdelta1* gene are frequently seen in class 1 integrons. Integrons are considered scarce in Gram-positive bacteria. Nevertheless, in the genus Corynebacterium, class 1 integrons have already been documented in *C. glutamicum*, *Corynebacterium ammoniagenes* and *Corynebacterium casei* [76] and, recently, a class 1 integron was identified in clinical urine isolate characterized as *Corynebacterium* spp. [77]. Additionally, according to the INTEGRALL database [78], in the genus *Corynebacterium,* there are class 1 integrons in *C. diphtheriae*, *C. amycolatum*, and *Corynebacterium efficiens*. However, class 1 integrons have not been described in *C. striatum*. Therefore, to evaluate the emergence or presence of integrons in *C. striatum*, greater epidemiological monitoring will be required, with studies focused on identifying the integrase gene *Int1* and its associated genes.

Fortunately, the glycopeptide vancomycin continues to show good results in the treatment of *C. striatum*. Although the *vanW* gene, which is associated with vancomycin resistance, was found in the genome of *C. striatum* 2308, this isolate proved to be susceptible to vancomycin [34]. However, vancomycin resistance in *Corynebacterium aquaticum* has been reported [39], and a case of infection due to vancomycin resistance in *Corynebacterium* spp. has also been documented [79].

Finally, based on searches of ARGs in databases like MEGARes [80] and ResFinder [81], we found several ARGs, indicating that the in silico resistome of *C. striatum* is larger (data not shown). This finding reflects the need to explore and characterize the resistome of *C. striatum* involving computational approaches of prediction methods and functional characterization.

## 4. Emergence of New *C. striatum* Clones

The studies compiled here indicate that *C. striatum* should not be underestimated, given that it is causing several types of infections. Nevertheless, despite the seeming virulence factors, recent studies revealed a mortal virulence of *C. striatum* in *Caenorhabditis elegans* [84,85], a nematode used as a simple animal model to study host–pathogen interactions [86]. Therefore, more attention must be paid to this pathogen in immunocompromised patients and the personnel assigned to this clinical picture.

The diversity of IS elements (Table 1) associated with ARGs in *C. striatum* highlights the plasticity of this bacterium in acquiring resistance genes and propagating as a persistent and pathogenic clone. Added to this, the ability to create biofilms in medical devices makes it a potential propagator of multidrug-resistant genomic elements. Thus, *C. striatum* may act as a potential reservoir to propagate resistance to other pathogenic bacteria.

*Corynebacterium* species can be detected in the clinical environment using biochemical techniques, commercial kits (such as API Coryne or Vitek 2) and recently MALDI-TOF (matrix-assisted laser desorption/ionization time-of-flight) [87]. Biochemical techniques do not distinguish among Corynebacterium species, so MALDI-TOF has become one of the most used clinical tools to detect pathogenic microorganisms, including *C. striatum* [3,87]. Whether or not molecular confirmation is needed at the species level is debatable; it has been recommended that the genus *Corynebacterium* spp. [88] should be identified at the species level. However, it is generally only necessary for drug treatment purposes to confirm if they belong to the genus *Corynebacterium* since the clinically relevant Corynebacterium species still show susceptibility to glycopeptides, such as vancomycin and linezolid [39,89,90].

Whether *C. striatum* could cause an epidemic is a question that has not yet been answered. Since it is part of the normal human microbiota, *C. striatum* has only primarily caused disease to date as an opportunistic pathogen. However, a recent phylogenetic study considering Chinese strains of *C. striatum* suggests that a lineage of *C. striatum* has been extended worldwide and could be a pandemic lineage [32]. Furthermore, due to the characteristics of its mobilome, the presence of persistent lineages, or the emergence of new ones, epidemiological monitoring of this microorganism and its potential related resistance factors are essential and urgent to be implemented.

## 5. Conclusions

In this review, based on the literature and searches in specialized databases, we have explored the antimicrobial resistance gene repertoire found in *C. striatum*. This resistome comprises chromosomal determinants and mainly acquired elements. Intrinsic resistance includes fluoroquinolone and daptomycin resistance via mutations on chromosomal genes. The increasing resistance and adaptation to daptomycin tolerance deserves special attention, which requires greater monitoring of its presence and the mechanisms that produce it. Many of the studies described here point out that a large part of the C. striatum resistance presents a genomic context similar to mobile elements such as insertion sequences and transposons mainly related to pTP10 plasmid.

The transposon Tn5432 stands out, which has been associated with the MLS phenotype and has insertion sequences along with different resistance genes. The ISs that compose this transposon have been identified in other bacteria and in isolates throughout the world. Although there are some similarities in the genomic context of ISs, we can observe some gene composition diversity and rearrangements in these regions. These findings suggest that insertion sequences are potential propagators of resistance at the interspecies level and play a fundamental role in the continuous formation and evolution of the resistome of *C. striatum*. Therefore, future challenges aim to systematically study the diversity of IS and its associated genes to understand the role of IS in the maintenance and dispersal of ARGs and its impact on the generation of lineages of concern of *C striatum*.

On the other hand, genes such as *qacEdelta1*, *sul*, *bla* and *merA* have not been associated with mobile genetic elements in *C. striatum*. However, considering their occurrence in other bacteria, they could be part of integrons or other genomic islands, making it necessary to carry out future studies focused on the genomic context of these genes. Moreover, although genes conferring resistance to vancomycin and linezolid have not been verified among nosocomial and community strains of *C. striatum*, the emergence of multidrug-resistant strains and acting as a reservoir for ARGs reveals a worrisome public health scenario. Therefore, in addition to the species-level confirmation, more significant efforts and attention should be dedicated to monitoring its occurrence and investigating its resistome.

This work uncovers the dynamics of antimicrobial resistance in *C. striatum* in the context of epidemiological factors, the ARGs repertoire, and its genomic localization. Furthermore, it is highlighted that the resistome of *C. striatum* is dynamic and in evident expansion. Consequently, it is emphasized that actions such as implementing genomic surveillance programs and studies on the mechanisms of antimicrobial resistance should be carried out. These measures would help to warn of the emergence of multidrug resistance lineages and prevent future public health problems.

## Figures and Tables

**Table 1:** Insertion sequences (IS) and composite transposons associated with resistance genes in *C. striatum* based on previous reports, ISfinder, and Tn registry web tools.

IS ^1^	Group ^1^	IS Family ^1^	Tn ^2^	Determinant Associated ^3^	Origin ^4^
IS6110	IS51	IS3		*tet(W), blaB*	*Mycobacterium tuberculosis*
IS3502				*tet(W), erm(X), mdtK*	*Corynebacterium jeikeium*
IS3501				*erm(X), tetA, tetB, tet(W), yheH*	*Corynebacterium jeikeium*
IS3504				*erm(X), aac(3)-XI, cmx*	*Corynebacterium diphtheriae*
IS1600				*aac(6′)-la*	*Shigella sonnei*
IS1206				*tet(W). erm(X), mdtK*	*Corynebacterium glutamicum*
IS407	IS407			*erm(X), tet(W)*	*Burkhoderia cepacia*
ISAar26	IS3			*sul1 ermE, cmx, strB, strA*	*Arthobacter arilaitensis*
IS3503	IS1249	IS256		*tet(W), blab*	*Corynebacterium jeikeium*
ISActu1				*tet(W), erm(X), mdtK*	*Actinomyces turicensis*
IS1249			Tn5432	*erm(X), tetA, tetB, tet(W), yheH*	*Corynebacterium striatum* ^5^
ISCre1				*aac(6′)-la*	*Corynebacterium reistens*
IS1250	ND		Tn5716	*strB, strA*	*Corynebacterium striatum*
IS6100	ND	IS6		*aac(6′)-la, aadA1*	*Mycobacterium fortuitum*
IS26	IS6		Tn5715	*aphA1-IAB, aph(3′)-I, strA, strB*	*Proteus vulgaris*
IS1628	ND			*aac(6′)-la*	*Corynebacterium glutamicum*
IS110	ND	IS110		*erm(X) tetAB, yheH*	*Streptomyces coelicolor*
ISCg9a	ND			*erm(X), aac(3)-XI*	*Corynebacterium glutamicum*
IS5564	ND	IS481	Tn5564	*cmxA, cml, erm(X) tetA/B, yheH*	*Corynebacterium striatum*
IS1513	ND	IS30	Tn5564	*cmx, cml*	*Corynebacterium striatum*

^1^ See ISfinder (https://www-is.biotoul.fr/, accessed 15 November 2020) for details of IS. ^2^ See Tn registry (http://transposon.lstmed.ac.uk/, accessed 17 November 2020) for further details. ^3^ Information is available from references in the text as well as from other references [13,30,32]. Seen in the same genomic context, which may include the presence of other IS. ^4^ First organism where the element was described according to ISfinder. ^5^ May appear as C. xerosis in various records due to misidentification.

**Table 2:** Genetic factors involved in drug resistance in *Corynebacterium striatum*.

Chemical Class	Drug ^1^	Mechanism of Action	Genetic Factor	Associated Function	Mechanism of Resistance	References
Fluoroquinolones	Levofloxacin Ciprofloxacin Moxifloxacin	Inhibits DNA gyrase and DNA topoisomerase	*gyrA*	Negatively supercoils closed circular double-stranded DNA	Alteration of drug target due to mutation	[46]
Phenicols	Chloramphenicol	Inhibits protein elongation due to 23S ribosomal subunit binding	*cmx*	Encoding of a specific efflux protein of chloramphenicol	Transports chloramphenicol out of the membrane	[35]
Macrolides	Erythromycin Clindamycin	Inhibits protein synthesis due to 50S ribosomal subunit binding	*erm(X)* *ermB*	23S ribosomal RNA methyltransferase	Alteration of drug target due to methylation	[35,47]
Tetracyclines	Tetracycline Doxycycline	Inhibits the initiation of translation by binding to 30S ribosomal subunit	*tetA, tetB*	Tetracycline efflux ABC transporter TetAB	Transports tetracyclines out of the membrane	[35]
			*tet(W)*	Tetracycline resistance ribosomal protection protein	Binds to the ribosome and inhibits the binding of tetracyclines	[16]
Beta-Lactams	Penicillin Ampicillin Cefazolin Cefotiam Cefotaxime Meropenem Cefotaxime Imipenem Oxacillin Ceftriaxone	Inhibits cell wall biosynthesis	*bla*	Beta-lactamase class A (serine hydrolase)	Alteration of the drug due to enzymatic modification	[20,33,47]
			*tetA* *tetB*	Tetracycline efflux ABC transporter TetAB	Transports beta-lactams out of the membrane	[35]
			*ampC*	Beta-lactamase class C	Alteration of the drug due to enzymatic modification	[47]
Sulfonamides	Sulfamethoxazole -/trimethoprim	Acts by blocking the synthesis of folic acid and inhibits growth	*sul1*	Dihydropteroate synthase	Antibiotic target replacement	[33]
	Trimethoprim		*ND*	ND	ND	
Aminoglycosides	Tobramycin Amikacin Streptomycin	Inhibits protein synthesis	*aph(3′)-Ic* *aph(3”)-Ib (strA)* *aph(6)-Id* *(strB)*	O-phosphotransferases	Catalyzes ATP-dependent phosphorylation of hydroxyl group	[47,82]
	Gentamicin Kanamycin		*aac(3)-XI*	N-acetyltransferases	Catalyzes acetyl CoA-dependent adenylation of an amino group	[83]
Lipopeptides	Daptomycin	The aggregation of daptomycin alters the curvature of the membrane, which creates holes that leak ions	*pgsA2*	Catalyzes the synthesis of phosphoglycerol (PG)	Inhibits membrane binding due to PG deficiency	[40]

^1^ Drug: The drugs were not necessarily verified by the authors cited in the reference column.
